# SAFIR-I: Design and Performance of a High-Rate Preclinical PET Insert for MRI

**DOI:** 10.3390/s21217037

**Published:** 2021-10-23

**Authors:** Pascal Bebié, Robert Becker, Volker Commichau, Jan Debus, Günther Dissertori, Lubomir Djambazov, Afroditi Eleftheriou, Jannis Fischer, Peter Fischer, Mikiko Ito, Parisa Khateri, Werner Lustermann, Christian Ritzer, Michael Ritzert, Ulf Röser, Charalampos Tsoumpas, Geoffrey Warnock, Bruno Weber, Matthias T. Wyss, Agnieszka Zagozdzinska-Bochenek

**Affiliations:** 1 Institute for Particle Physics and Astrophysics, ETH Zürich, 8093 Zürich, Switzerland; beckerr@phys.ethz.ch (R.B.); vcommichau@gmx.de (V.C.); jdebus@ethz.ch (J.D.); disserto@ethz.ch (G.D.); Lubomir.Djambazov@cern.ch (L.D.); jannis.fischer@phys.ethz.ch (J.F.); mikiko.ito@lge.com (M.I.); pkhateri@phys.ethz.ch (P.K.); werner.lustermann@cern.ch (W.L.); critzer@phys.ethz.ch (C.R.); roeser@phys.ethz.ch (U.R.); a.zago@phys.ethz.ch (A.Z.-B.); 2 Institute of Pharmacology and Toxicology, University of Zürich, 8057 Zürich, Switzerland; afroditi.eleftheriou@pharma.uzh.ch (A.E.); geoffreyiain.warnock@uzh.ch (G.W.); bweber@pharma.uzh.ch (B.W.); mwyss@pharma.uzh.ch (M.T.W.); 3 Institute of Computer Engineering, Heidelberg University, 69120 Heidelberg, Germany; peter.fischer@ziti.uni-heidelberg.de (P.F.); michael.ritzert@ziti.uni-heidelberg.de (M.R.); 4 Leeds Institute of Cardiovascular and Metabolic Medicine, University of Leeds, Leeds LS2 9JT, UK; c.tsoumpas@leeds.ac.uk

**Keywords:** PET detector, PET insert, MRI, preclinical PET/MRI, dynamic PET/MR imaging, high frame rates, 5 s time frames, temporal resolution

## Abstract

(1) Background: Small Animal Fast Insert for MRI detector I (SAFIR-I) is a preclinical Positron Emission Tomography (PET) insert for the Bruker BioSpec 70/30 Ultra Shield Refrigerated (USR) preclinical 7 T Magnetic Resonance Imaging (MRI) system. It is designed explicitly for high-rate kinetic studies in mice and rats with injected activities reaching 500 MBq, enabling truly simultaneous quantitative PET and Magnetic Resonance (MR) imaging with time frames of a few seconds in length. (2) Methods: SAFIR-I has an axial field of view of 54.2 mm and an inner diameter of 114 mm. It employs Lutetium Yttrium OxyorthoSilicate (LYSO) crystals and Multi Pixel Photon Counter (MPPC) arrays. The Position-Energy-Timing Application Specific Integrated Circuit, version 6, Single Ended (PETA6SE) digitizes the MPPC signals and provides time stamps and energy information. (3) Results: SAFIR-I is MR-compatible. The system’s Coincidence Resolving Time (CRT) and energy resolution are between (209.0±0.3) ps and (12.41±0.02)% Full Width at Half Maximum (FWHM) at low activity and (326.89±0.12) ps and (20.630±0.011)% FWHM at 550 MBq, respectively. The peak sensitivity is ∼1.6%. The excellent performance facilitated the successful execution of first in vivo rat studies beyond 300 MBq. Based on features visible in the acquired images, we estimate the spatial resolution to be ∼2 mm in the center of the Field Of View (FOV). (4) Conclusion: The SAFIR-I PET insert provides excellent performance, permitting simultaneous in vivo small animal PET/MR image acquisitions with time frames of a few seconds in length at activities of up to 500 MBq.

## 1. Introduction

The aim of the Small Animal Fast Insert for MRI (SAFIR) project is the construction of a Positron Emission Tomography (PET) insert for Magnetic Resonance Imaging (MRI) with unprecedented temporal resolution, providing high-quality imaging within less than 5 s, truly simultaneous with MRI. The ultimate goal is a system with 145 mm axial coverage, facilitating preclinical fast quantitative kinetic PET/MRI research in mice and rats for injected dose rates of about 500 MBq with full-body imaging.

PET technology has been around for more than 60 years and is well established in clinical and preclinical applications, including the combination of PET with Computed Tomography (CT) and, more recently, with MRI [1]. Nevertheless, it is still advancing continuously even today. An overview of small animal PET technologies, including multimodality PET systems and available image reconstruction and analysis tools, both in research and commercial products, is given in [2]. Recent developments in preclinical PET/MRI instrumentation are briefly summarized in the introduction of [3] and reviewed in detail in [4]. Since the very beginning, advances in PET instrumentation were linked to progress in key technologies and to new ideas, be it brighter and faster scintillators, magnetic field compatible photo-sensors or Time-of-Flight (TOF)-PET. For example, the commercial availability of Avalanche Photo Diodes (APDs) enabled the construction of the first PET insert integrating the photo-sensors into the bore of an MRI magnet [5], thanks to their insensitivity to magnetic fields. Practically all modern clinical and preclinical systems use Silicon Photomultipliers (SiPMs), alias Multi Pixel Photon Counters (MPPCs), or their digital counter parts digital SiPMs (dSiPMs) [6], combined with lutetium-based scintillators such as Lutetium OxyorthoSilicate (LSO), Lutetium Yttrium OxyorthoSilicate (LYSO) or Lutetium Gadolinium OxyorthoSilicate (LGSO). Other scintillator materials are under investigation and might improve detector performance in the years to come. Their fast timing response enabled TOF-PET to become a standard in clinical PET systems, thereby boosting the Signal-to-Noise Ratio (SNR) [7,8]. The SiPM-based Siemens Biograph Vision PET/CT system reaches 210 ps Full Width at Half Maximum (FWHM) Coincidence Resolving Time (CRT) [9]. Moreover there is a push towards reaching 10 ps CRT [10] improving SNR further and achieving “reconstruction free” PET.

In many studies spatial resolution is of high importance. Preclinical PET/MRI scanners have reached sub-millimeter resolution, shown in examples [3,11,12,13,14,15]. A recent summary of design and performance of 38 preclinical systems [11] quotes spatial resolutions between 0.6 mm and 3.0 mm with a median of 1.42 mm [16]. Eight of the systems use Depth Of Interaction (DOI) information. The reported energy resolutions range from 11.7% to 26% (We excluded the quoted energy resolution of the LFER scanner, because we suppose the numbers are misprinted, being identical to the quoted peak-sensitivity values. Moreover, the referenced paper [17] does not quote a value for the energy resolution) and the timing resolutions from 409 ps to 4.1 ns. Sensitivity is another important aspect of PET instrumentation. The higher it is the lower the injected dose may be or the faster the required number of counts can be recorded to achieve a desired image quality. With the development of the first full body PET/CT the Explorer project achieved a huge sensitivity increase for clinical applications [18], which can be exploited for the benefit of patients.

The SAFIR project (Project webpage: https://dissertori-group.ethz.ch/research/detector-rd-and-applications-in-biomedical-imaging/safir.html, accessed on 21 October 2021) is not trying to push back the frontier of spatial resolution; its main aim is to significantly reduce image acquisition times. It will open up new opportunities with respect to SNR, operating at activities more than 10× higher than in typical preclinical systems, for applications where injected activities of 500 MBq are acceptable. This requires state of the art CRT values be reached, that are comparable to the ones achieved in clinical TOF/PET systems. Driven by this motivation, SAFIR is pushing the frontier of PET electronics integration into an MRI system. Compared with other preclinical PET inserts, Small Animal Fast Insert for MRI detector I (SAFIR-I) is a unique device, being the first insert with analog SiPMs to integrate the entire readout electronics, including DC-DC power conversion (see [19]), into the extremely limited space inside the bore of a preclinical MRI system [20,21]. In total 144 readout Application Specific Integrated Circuits (ASICs), 61 DC-DC converters and twelve Gigabit Ethernet transceivers are used to individually digitize the signals of 4320 channels.

The specifications for SAFIR were established together with the research group of Prof. B. Weber at the University of Zurich (UZH) Institute of Pharmacology and Toxicology. At the heart of the project’s inception was the search for a novel PET insert to an existing Bruker BioSpec 70/30 Ultra Shield Refrigerated (USR) 7 T MRI system, facilitating preclinical studies beyond the capabilities of presently available commercial PET/MRI devices. Examples include studies on cerebral and cardiac energy metabolism simultaneously using hyperpolarization- and radio-labeled pyruvate, where dynamic PET data could conceivably be used to improve MRI-based kinetic modelling of non-linear transport mechanisms. Furthermore, quantitative non-invasive PET with excellent temporal resolution could be used together with MRI for an absolute quantification of Cerebral Blood Flow (CBF). This would in turn facilitate studies of neurodegenerative diseases involving CBF deficits such as Alzheimer’s disease [22], as well as microvascular impairment in the context of ischemic strokes, specifically reperfusion failure also known as no-reflow phenomenon [23,24,25].

With this research in mind, a cutting-edge PET/MRI system should enable not only truly simultaneous acquisition of images with both modalities but also be capable of capturing fast dynamics of molecular readouts, which are impractical to obtain with separate devices. To fulfill this condition by delivering images of suitable quality within a few seconds, the PET system needs to be capable of high-speed data acquisition at injected dose rates of ∼ 500 MBq to compensate for the reduced statistics in short time frames, to maintain a suitable image quality. Tied to the image quality is a requirement for a supreme CRT, the shorter the better, allowing for a short Coincidence Timing Window (CTW) limiting the number of random coincidences. We aimed at ∼ 300 ps (FWHM) [26]. Furthermore, the system has to achieve a spatial resolution in the center of the Field Of View (FOV) of ∼ 2 mm. It is worth noting the trade-off between spatial and temporal resolution. A reduction in the voxel size by a factor of two results in an eight-fold increase in measurement time to maintain quantitative accuracy in the PET image. Hence, we aim at a moderate spatial resolution, compared to state of the art PET systems, adequate for the targeted research. With regards to quantitative kinetic modelling of fluxes of metabolic reactions, the PET system additionally needs to be capable of delivering quantitative dose rate concentration data.

In pursuit of this target, SAFIR designed a prototype [27] which was in turn extended to SAFIR-I described in this paper, by increasing the axial FOV by 50% to 54.4 mm. The fundamental performance measurements of SAFIR-I are summarized in Section 2 and Section 3.

To the best of our knowledge, there is no other preclinical PET insert available which has been tested at decay rates of 500 MBq and could provide the count rate statistics envisaged by the SAFIR project. In particular, we are aiming at a Noise-Equivalent Count Rate (NECR), determined according to NEMA Standards Publication NU 4-2008 (NEMA-NU4) [28], which peaks only far beyond 500 MBq. In [16] peak NECR, values for mouse ranging from 16.9 kcps to 1670 kcps with a median of 204 kcps and for rat ranging from 12.8 kcps to 592.0 kcps with a median of 156 kcps are reported (With varying energy windows and excluding the Mini-EXPLORER [29] and Mini-EXPLORER II [30] NECR data because both systems are targeting primates instead of small rodents). The maxima are reached by the Siemens Inveon stand-alone PET detector with a peak sensitivity of 6.72% [31]. The SAFIR prototype system with a peak sensitivity of 1.06% reached 799 kcps at 537 MBq for mouse and 121 kcps at 624 MBq for rat, without reaching the NECR peak in either case [32]. The Bruker PET Insert, which was designed explicitly for use inside the Bruker BioSpec 70/30 USR MRI instrument, has been observed to reach maximal NECR values of 486 kcps and 249 kcps, at activities of 22.58 MBq and 22.85 MBq, for mouse and rat phantoms, respectively, [3].

## 2. Materials and Methods

### 2.1. Detector Concept

SAFIR-I is a custom designed preclinical PET insert with an axial FOV of 54.2 mm, which fits into the Bruker BioSpec 70/30 USR 7 T MRI system. It has an annular cylindrical shape, with an outer diameter of 198 mm and a length of ∼ 1 m, making it compatible with the bore dimensions of the Bruker MRI apparatus and its standard Bruker BGA-20S HP gradient system. Further, SAFIR-I’s inner diameter of 114 mm enables the installation of Bruker whole body resonators inside (e.g., Bruker BioSpin RF RES 300 1H 112/086 QSN TO AD and RF RES 300 1H 112/072 QSN TR M).

**Figure 1:**
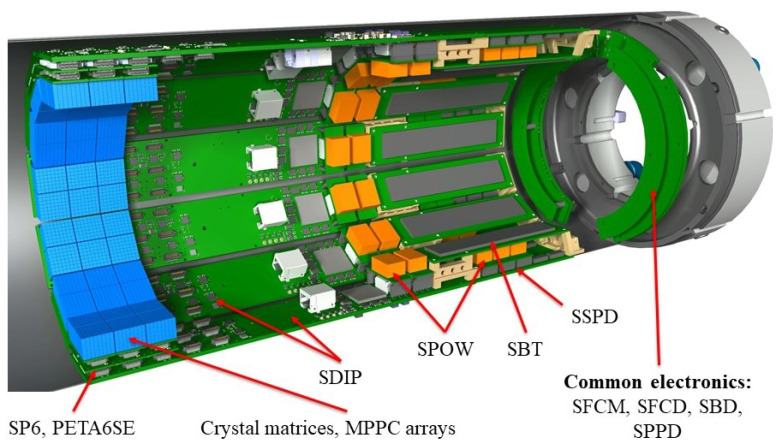
View of the SAFIR-I detector components.

The SAFIR-I insert employs 4320 Lu${}_{2(1-x)}$Y${}_{2x}$SiO${}_{5}$ Lutetium Yttrium OxyorthoSilicate (LYSO) crystals supplied by Meishan Boya Advanced Materials, which are coupled one-to-one to Hamamatsu S13361-2050AE-08 SPL0 (The employed MPPC arrays are a customized version of Hamamatsu S13361-2050AE-08 featuring common anode instead of common cathode) Multi Pixel Photon Counter (MPPC) arrays and arranged along a dodecagon (see Figure 1). The insert’s electronics are highly integrated, featuring the Position-Energy-Timing Application Specific Integrated Circuit, version 6, Single Ended (PETA6SE) [33,34] as a readout chip. All parts required for the processing and digitization of MPPC signals as well as components used for power conversion and conditioning are integrated into the insert and operate inside the 7T magnetic field of the MRI system. This minimizes external services and interconnections required for the operation of the device. Forced air flow cooling removes the dissipated power.

Internally SAFIR-I is divided into 12 identical sectors, hosting the Detector Head Modules (DHMs) (see Section 2.3) and all electronic components required for their operation and readout. The electronic components for power distribution and synchronization signal distribution, which are common to all sectors, are located at one end of the cylinder. Moreover, all services pass through this side, namely power cables and optical fibers for data transmission and control. The other end of the cylinder features an annular chamber promoting even distribution of incoming air flow to all sectors. Figure 1 provides an overview of the major detector components and their arrangement. All components are housed inside a carbon fiber support structure, which simultaneously provides Radio Frequency (RF) screening (Several groups, such as the Schug group at RWTH Aachen, have already used carbon fiber for RF screening in MR applications to great effect [35,36]) (see details below), as well as means to connect the insert to air cooling manifolds on both ends.

Peripheral hardware that complete the system include external low voltage power supplies, a Data Acquisition (DAQ) and control computer, dedicated DAQ and control software and an air cooling system. These components are located in a service room, about 10 m away from the insert, and all connections enter the Faraday cage of the MRI system through a filter plate.

**Figure 2:**
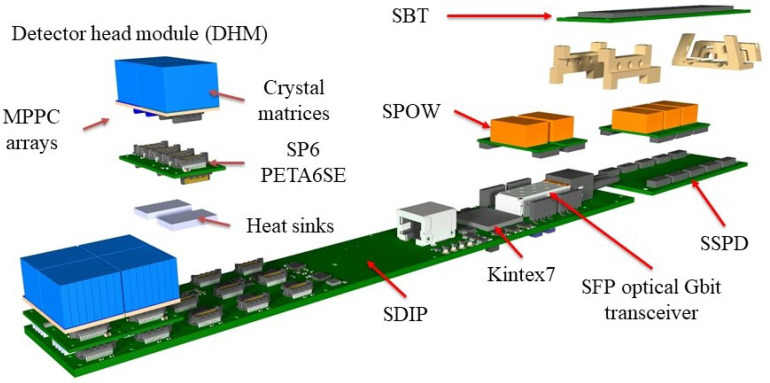
An exploded view of the casket electronics.

The DHMs, together with associated readout components of each sector, are individually separated and shielded from the rest of the detector (including the other sectors) by embedding them into a closed carbon fiber housing, the casket. Specifically, each casket contains the following elements (see Figure 2):

- The core of each sector is the SAFIR Digital Interface Board PETA (SDIP), which has connections to the DHMs, a SAFIR Bias and Temperature (SBT) Printed Circuit Board (PCB) and the DAQ computer via a commercial optical Gigabit Small Form-factor Pluggable (SFP) transceiver module.
- Three identical DHMs incorporating the MPPC arrays (two per module) and the PETA6SEs (four per module). The DHMs interconnect MPPCs, SAFIR PETA6 Board (SP6) and SDIP.
- One SBT board, providing bias voltage to the MPPCs on all three DHMs.
- Five DC-DC SAFIR Power Converters (SPOWs), powering the three DHMs (one converter each) and the SDIP (two converters).
- One SAFIR Secondary Power Distribution (SSPD) board; a passive board hosting the SPOWs powering DHMs.
- A mechanical support structure for the different components.
- All necessary cabling.

Outside of the individual caskets, the following electronics boards service the caskets and are common to all 12 sectors:

- SAFIR Fast Control Master (SFCM) and SAFIR Fast Control Distribution (SFCD): Control and synchronization boards.
- SAFIR Primary Power Distribution (SPPD): Power distribution for SDIPs and DHMs.
- SAFIR Bias Distribution (SBD): Power distribution for the SBTs.

In the following, we detail relevant features of all components.

### 2.2. Mechanical Structure and Cooling

#### 2.2.1. Mechanical Structure

The mechanical structure of SAFIR-I was designed to meet the four following objectives:

1. Provide a solid and precise structure for exact crystal alignment.
2. Offer reliable RF shielding of SAFIR-I from the MRI system, and vice versa.
3. Facilitate good air cooling.
4. Be modular and practical to work on.

**Figure 3:**
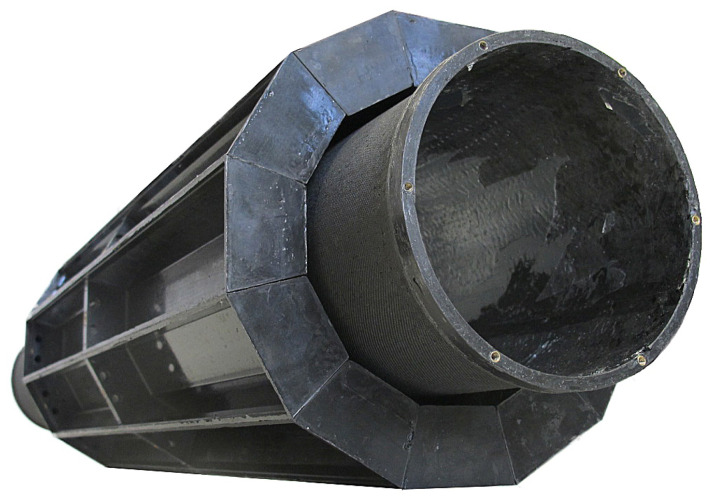
Carbon fiber structure with inner cylinder and 12 caskets.

The structure centers on a carbon fiber inner cylinder where precision molded flanges align 12 carbon fiber caskets radially to form the complete detector ring with its 12 sections (see Figure 3).

Each casket is an individually shielded carbon/graphite composite box closable with its own lid. The inner cylinder extends fore and aft of the casket array, making space for common power and signal distribution electronics as well as cabling, and allowing the cooling air flow to stabilize prior to entry and exit. Carbon/graphite composite end-flanges cap and shield the detector at both ends. Additional hollow polyethylene rings mounted to the carbon end-flanges on either side manifold the incoming and exiting cooling air, and contain six cam/piston mechanisms (three on each side) to secure SAFIR-I in the inner bore of the MRI system. Additional recesses in the front ring (on the animal bed side) allow for precise positioning within the MRI system by means of alignment pins. A cylindrical carbon/graphite composite outer shell shields, protects, and closes the detector.

Precision was of paramount importance for the exact alignment of the detector head. Specifically, this included the internal alignment of crystal matrices inside the casket, precise manufacture of each casket’s pair of interface holes (3 mm H7), and reciprocally, precise inner cylinder flange holes were needed to align the 12 caskets to form a precise detector ring.

Inside the casket, crystal matrix alignment is given by the PCB connector placement precision (<0.05 mm), the casket groove that the matrices fit into, and by the two molded 3 mm H7 holes that secure the SDIP board into the casket. The casket matrix slot width is measured to be 0.03 mm, and the axial distance between holes is ±0.05 mm (Computerized Numerical Control (CNC) driven). Next, each casket is mounted on the inner cylinder via precision holes molded into two flanges. Thus, the precision of the casket mounting onto the inner cylinder depends on the precision of the machined mandrel and 12 sided flange molds: After lathing the exact 114 mm diameter of the mandrel, it was clamped into a parting head on a milling machine. All caskets fit from the onset, each having a mere 0.1 mm clearance between them, confirming a cumulative precision of better than ±0.05 mm in any direction.

SAFIR-I itself is aligned via two pins/holes in the air inlet end flange and a holder sporting these two pins hard-mounted inside the MRI machine. Furthermore, the centers of the two systems are aligned.

#### 2.2.2. Carbon Fiber Structure Building Techniques

**Figure 4:**
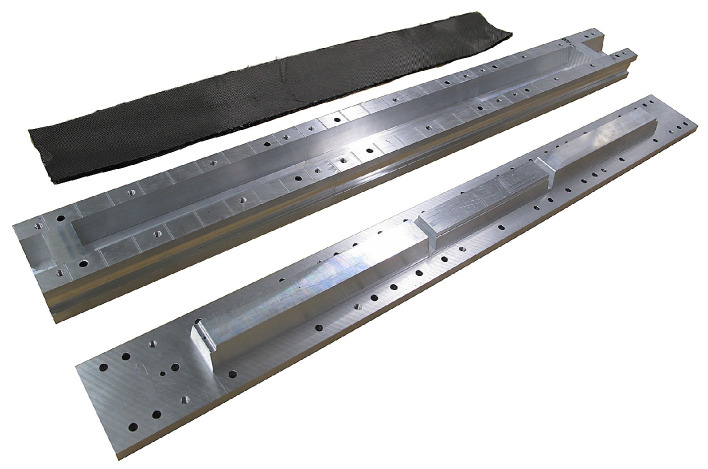
Casket molding (from the top): Carbon fiber sheets, heatable female mold and male mold.

**Figure 5:**
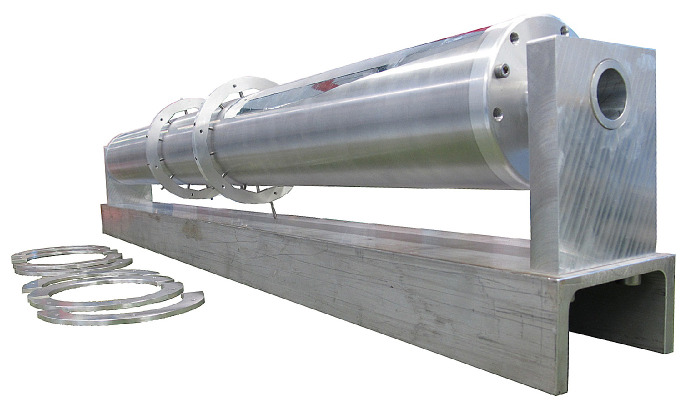
Mandrel for the fabrication of the inner carbon fiber cylinder.

To offer some measure of RF shielding, all composite components were manufactured in-house using non-standard building techniques. Enclosures were packed with several layers of cloth at 90-0 and 45-45 degree fiber orientations, impregnated and cured using graphite loaded epoxy. The graphite loaded epoxy mixture contained: R&G Epoxy Resin L, cured via Hardener CL with a mixing ratio of 10:3. Additionally, 20% (by weight) graphite powder was blended into the mixture prior to lamination and molding. Caskets and their covers were press-molded using custom aluminium molds: The epoxy/graphite mixture combined with 6 mm chopped fiber created a paste that was used to fill all of the mold’s pockets, i.e., the mid-wall and steps in the side walls. Two epoxy/graphite impregnated layers of Swiss-composites Spread e442 160 g·$m^{-2}$ and another two layers of Biaxial ± 45° 200 g·$m^{-2}$ cloth were hand laminated onto the male mold, before slowly inserting into the female molds (see Figure 4). The mold is bolted together and allowed to cure for 24 h at 70 °C. Casket covers were molded in an analogous way, the only difference being that the epoxy/graphite laden carbon cloth was laid into the heated female mold prior to pressing with the male mold. The inner cylinder and outer cylindrical covers were manufactured via hand-lay-up and vacuum bagging about precision mandrels (see Figure 5). The inner cylinder was wrapped with three layers of cloth with 0-90, 45-45, and again 90-0 fiber orientation; the outer cylinder covers with two thicker layers of 45-45 and 0-90 fiber orientation cloth. The mold mandrel is hollow inside and end caps sport an inlet and outlet for hot air to flow inside. The lay-up was cured for 24 h at 70 °C, loosely insulating the outside with flexible insulation (cloth). Lastly, end caps were press-molded into a female aluminium mold, using pre-cut carbon cloth and 6 mm chopped carbon mixed into the epoxy/graphite mixture and also cured for 24 h at 70 °C.

The RF shielding of the carbon/graphite composite structure (compare to [37]) is complemented by multi-layer copper-Kapton shields around the central 20 cm section of the inner cylinder and copper shields on the back of the DHM region, between the electronics and the casket lid.

#### 2.2.3. SAFIR-I Air Cooling System

Keeping the crystal matrices and MPPCs cool at a steady temperature was the main focus of SAFIR-I’s cooling system, while simultaneously keeping the readout and power electronics at acceptable operating temperatures was a given. To make SAFIR-I straightforward and safe to use for novice users, cooling was to be simple and intuitive to connect and operate.

SAFIR-I is air cooled inside a closed loop circuit with a recirculating air flow. A side channel blower sucks air through SAFIR-I and vents the exhaust into a room where an air conditioner chills the heated air back down to ∼16 °C, prior to it being ducted back to SAFIR-I’s inlet tubing. Bulk cooling capacity of this setup is in the order of 1.2 kW, which takes care of the heat load created by SAFIR-I (≲550 W at 500 MBq), compression heating of the cooling air inside the blower (600 W) and losses through the insulated circuit (estimated few tens of watts).

Caskets were designed with a ducted air entry at the crystal matrix end, and a large exit at the electronics end. The total pressure drop across SAFIR-I was measured at ∼65 mbar.

In the unexpected event of sudden cooling failure, the system is protected by temperature sensors on all DHMs that trigger an automatic shut-off of modules at temperatures ∼10 °C above normal operating level.

### 2.3. Detector Head Module

**Figure 6:**
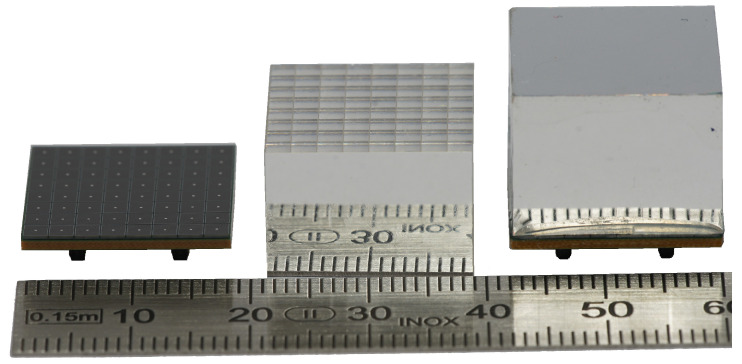
Image of SAFIR-I’s LYSO crystal assembly. The front reflective foil has been removed from one crystal block to showcase the crystal matrix. Crystal blocks (in the middle) are glued onto 8 × 8 Hamamatsu MPPC arrays (on the left) to form the completed assembly (on the right).

The DHMs comprise the crystal matrices, MPPC arrays and the readout Application Specific Integrated Circuits (ASICs) PETA6SE on SP6 carrier PCBs (see Section 2.4.1). We use Meishan Boya Advanced Materials Co., Ltd. (former Sichuan Tianle Photonics) LYSO crystals with dimensions of 2.12 mm × 2.12 mm × 13.0 mm, polished on all six sides. The crystals are arranged into two types of matrices, one with 8 × 8 and one with 7 × 8 crystals. Reflector foils (3M Enhanced Specular Reflector, thickness 0.065 mm) are used as separators between crystals and on all side faces except the one coupling to the photo sensors. The matrices are assembled by gluing crystals and reflector foils together, where the chosen dimensions of the components and adhesive layers allow a 2.2 mm pitch between crystal centers (see Figure 6). Crystal matrices were assembled by Meishan Boya Advanced Materials Co., Ltd., Meishan, China.

The photo sensors are Hamamatsu Photonics K.K. 8 × 8 MPPC arrays, type S13361-2050AE-08 SPL0, composed of 2.0 mm × 2.0 mm SiPM sensors with a pitch of 2.2 mm (see Figure 6), that we operate at 6 V overvoltage. We compared the MPPCs at several operating voltages, and found that there was no significant gain from more than 6 V overvoltage [38]. The MPPCs feature common anode readout, which allows connecting all of them to a common reverse (i.e., negative) bias voltage; the cathodes of the arrays are connected directly to the PETA6SE ASICs inputs, providing a negative input signal as required.

Crystal matrix and MPPC are precisely aligned with respect to each other for exact pitch-matching in a one-to-one coupling of crystal and sensor and bonded in place with an optical adhesive. This operation was carried out by Hamamatsu Photonics K.K. We chose a one-to-one coupling over a light sharing approach to minimize the count rate per channel. This minimizes possible pile-up of scintillation photons in the photo sensor, which could lead to degraded timing or energy resolution and potentially to the loss of hits in the processing chain of the PETA6SE. However, this method results in a large number of readout channels, complicating the electronics design.

### 2.4. Electronics

**Figure 7:**
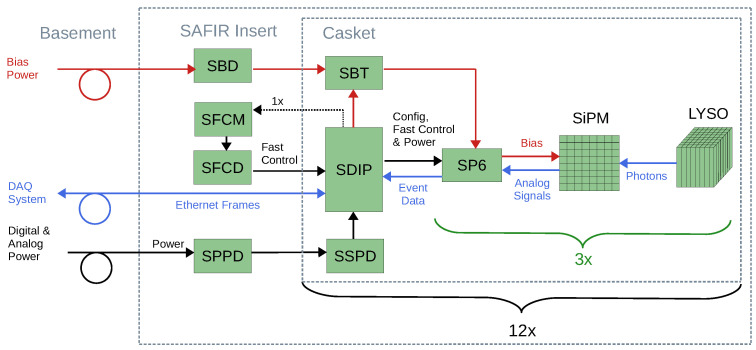
Schematic depicting the interaction of components within SAFIR-I. Each of the 12 SDIPs supports one SBT and three DHMs, consisting of LYSO scintillation crystals, MPPC SiPM arrays and SP6 PCB’s. These components are all connected to common supporting PCB’s in the detector’s end-flange. Bias, analog and digital power supplies as well as the DAQ system PC are located outside the insert within the service room in the basement.

SAFIR-I’s electronic components are custom designed and highly interconnected. A simplified block diagram of their functionality and interactions is shown in Figure 7. The following section describes the components individually.

#### 2.4.1. PETA6SE Signal Processing

The signals from the MPPC arrays are processed by the PETA6SE. This ASIC, provided by the University of Heidelberg, comprises 36 single-ended analog input channels, two serial output links as well as clock and control inputs. We do not use all of the input channels because of the chosen crystal matrices with 64 and 56 elements. It is worth noting that the chip internally features two identical halves of 18 channels, with separate Time-to-Digital Converters (TDCs)—one per half—and separate readout links.

Each ASIC channel features a front-end amplifier, followed by two parallel branches, a timing branch with a fast discriminator triggering the TDC and an energy branch connected to a Charge-to-Digital Converter (QDC) with adjustable gain. The acquisition in each channel is controlled by a finite state machine. Once an incoming signal pulse surpasses the timing threshold, it initiates the state machine and simultaneously records a time-stamp—consisting of a ‘fine’ counter value (50 ps nominal bin width, 5 bit length) and a ‘coarse’ counter value (1.6 ns bin width, 15 bit length)—from a continuously running oscillator serving as TDC; all TDCs inside SAFIR-I are running synchronously. Moreover, it triggers a 9 bit QDC, which integrates the incoming charge, registering it as a ‘hit’ if it surpasses a configurable energy threshold. The digitized information is stored in a register. Data readout is launched by an external signal which copies the data of all used channels of a chip half into a shift register for sequential readout. This simultaneously resets the state machines, which previously had a ‘hit’, back to idle, enabling the registration of the next ‘hit’.

The energy information is obtained by discharging a capacitor integrating the input charge (one per channel) with a constant current. The discharge speed and the start of the discharge are programmable. Moreover, discharging starts prior to the end of charge collection, speeding up the conversion process. The discharge time depends on the input charge. We estimate an average discharge time of ∼384 ns for a 511 keV interaction. The total average conversion time is 440 ns, which was estimated using test triggers; a feature of the PETA6SE making it possible to bypass the thresholds and artificially trigger the readout of all channels. The same feature is also used in our system to verify proper operation of the data readout of all ASIC channels and as part of the calibration routine described in Section 2.6.1.

The DC level of each input of the PETA6SE can be adjusted by up to 800 mV. However, we do not use this capability due to the excellent uniformity of the MPPC arrays. We set all PETA6SE bias voltage Digital-to-Analog Converters (DACs) identically, to 300 mV.

**Figure 8:**
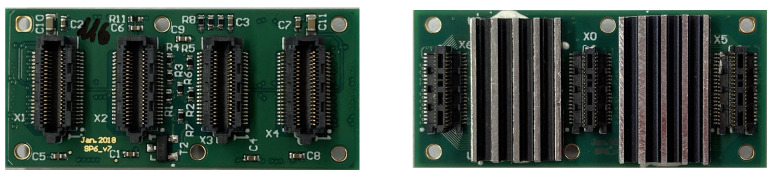
Image depicting the top and bottom side of the SP6. Four PETA6SE ASICs are located beneath aluminium heat sinks on the bottom side of the PCB, along with three male SAMTEC connectors (ST4-20-1.00-L-D-P-TR) which couple it to the SDIP, while the four female SAMTEC connectors (SS4-20-3.50-L-D-K-TR) on the top side are used to mount the MPPCs.

The PETA6SE is prepared for flip-chip bonding. The naked die is 6 mm × 5 mm small with 109 solder balls on a 500 μm grid. Integrated in the DHM, four PETA6SEs are mounted on one SP6 PCB (shown in Figure 8) interfacing MPPC arrays and SDIP through SAMTEC SS4/ST4 series connectors of 4.5 mm mating height. To promote efficient air cooling, the SP6 hosts aluminium heat sinks, which are permanently attached to the PETA6SEs by means of a thermal adhesive.

#### 2.4.2. Digital Interface Board

**Figure 9:**
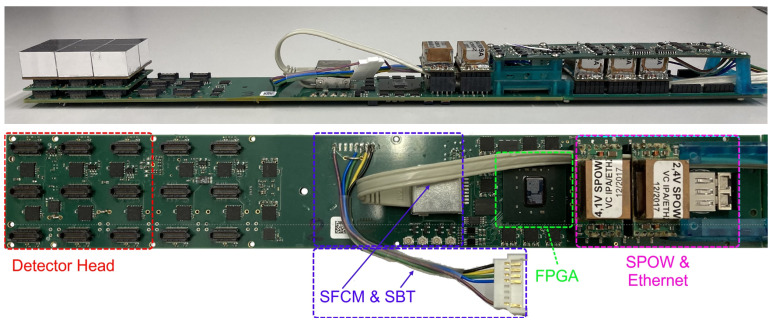
The SDIP. The upper image depicts the full casket assembly (side view), including the detector head on the left, as well as the SSPD and SBT PCB’s on the right. Below is a birds-eye-view of the SDIP itself. Central component is a Kintex-7 FPGA (marked in green), connected to the DHMs via SAMTEC connectors (red), the SFCM and SBT via cabling (blue), and the DAQ system via an SFP module located under SPOWs (pink).

The SDIP (depicted in Figure 9) is responsible for data readout, ASIC configuration and interfacing between the SP6 and further PCBs, as well as ultimately to the DAQ computer. At its core, a *Xilinx Kintex-7 XC7K70T* Field-Programmable Gate Array (FPGA) acquires event data from the PETA6SE ASIC using a serial protocol running at 280 MHz readout frequency. This FPGA provides an ’epoch’ counter (1.6 μs bin width, 32 bit length) for each received event, which is used as an extension of the time stamp to account for the wrap-around of the PETA6SE coarse counter. The wrap-around of the epoch counter (once every 7040 s) is in turn corrected for by dedicated software (see Section 2.6). The FPGA and thus the SDIP is connected to the DAQ computer by means of a commercial Avago AFBR-57R5APZ optical gigabit-transceiver SFP type module.

Events of all three DHMs installed per SDIP are bundled and transmitted via a 1 Gbit·$s^{-1}$ optical link using the User Datagram-Protocol (UDP) Ethernet protocol. The link allows communication and control from the DAQ computer to the SDIP, which in turn controls and configures the connected PETA6SE.

Furthermore, a MicroBlaze^TM^ (Xilinx, San Jose, CA, USA) softcore micro-controller instantiated within the FPGA accepts slow control packages via Ethernet and is used to facilitate communication to the SBT, as well as reading out several on board temperature sensors via the I^2^C interface and transmitting their data to the DAQ computer.

The SDIP carries two SPOW power converters, powering intermediate rails with 4.1 V and 2.4 V. Several Low Dropout Regulators (LDOs) powered from these rails provide all voltages required by the on-board components. The power consumed by the DHMs (6 W [4.8 W SP6, 1.2 W MPPC at 500 MBq]) originates from SPOW converters mounted on the SSPD board (see Section 2.4.5), to which the SDIP connects.

Four control signals common to all SDIPs arrive from the SFCM and SFCD PCBs (see Section 2.4.3) via an RJ45 port. These signals include the 31.25 MHz system clock, a fast reset, a trigger signal to activate the PETA6SE’s test trigger feature and a power enable signal. They are distributed on the SDIP to all PETA6SE using clock distribution buffers. The system clock is cleaned by a programmable jitter attenuator, providing the 625 MHz Phase-Locked-Loop (PLL) reference clock to the PETA6SE and a 156.25 MHz synchronization clock to the FPGA. All PETA6SE fine and course counters run in phase with the PLL reference clock, the epoch counter in the FPGA runs in phase with the synchronization clock. Upon a reset signal all above mentioned timing counters in the PETA6SEs and in the FPGAs are zeroed. The power enable signal starts an on-board power sequencer, which activates the SDIP’s LDOs in the order required by the FPGA.

#### 2.4.3. Synchronization and Fast Control

**Figure 10:**
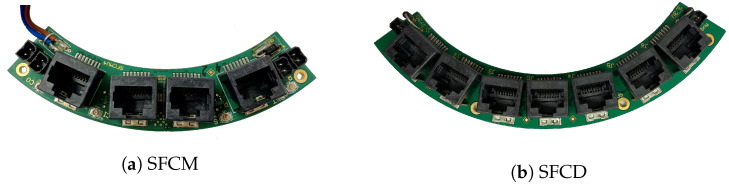
(**a**) The SFCM PCB, featuring four RJ45 ports for fast signal distribution. Three coaxial cable connections receive fast signals from a master board, which are distributed to two SFCD PCBs. (**b**) The SFCD features one input connector in the center and six outputs to distribute the signals from the SFCM to the SDIPs.

The four control signals mentioned in Section 2.4.2 are generated by the SFCM PCB (see Figure 10a) and distributed to all 12 SDIPs via two SFCD (see Figure 10b) using shielded flatband CAT-6 cables. With the exception of the power enable, these signals are required to have a phase jitter not larger than 30 ps, assuring that they do not significantly contribute to the systems CRT. This also ensures synchronized time stamps across the entire system since the fine, coarse and epoch counters are ultimately derived from the system clock and reset.

A 31.25 MHz crystal oscillator provides the system clock, while the other signals generated on the SFCM are triggered via coaxial cables from a single ‘master’ SDIP following a software command.

The power enable signal controls the power-up and power-down of the entire detector. When supplying power the system, the first enable signal is active by default. Delay elements on the SFCDs then set one power enable signal after the other. This reduces the power consumption of the detector at start-up and assures that all FPGAs boot properly. Detector shut down is performed sequentially when the master SDIP sends a disable signal to the SFCM.

A SPOW module located on the bottom side of the SFCM PCB powers the SFCM and both SFCDs.

#### 2.4.4. Bias Voltage System

**Figure 11:**
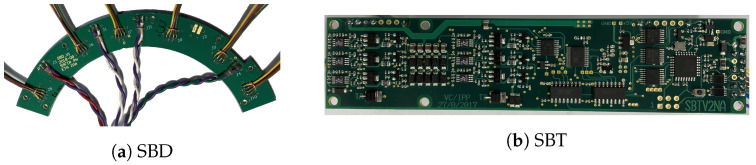
(**a**) Starting from the bias power supplies we use two SBDs to supply the required input voltages to all SBTs. The SBDs connects to the bias power supplies via four twisted-pair cables (at the bottom of the image) and serves six SBTs. (**b**) The SBT regulates up to six bias voltages between −50 V and −60 V.

Every sector contains one SBT board (depicted in Figure 11b). It regulates the bias voltages for the three DHMs connected. The two MPPCs per DHM are operated with the same bias voltage. The pairs are matched with respect to the their nominal operation voltages of ∼58 V. The largest difference between any two MPPCs on one DHM is 100 mV. The SBT features six bias voltage channels (three of them used in SAFIR-I). Its reference potential is floated to a stable negative potential of −50 V—the shift voltage—with respect to SAFIR-I’s reference potential. On top of the shift-voltage, the output voltages are regulated in a range from 0 V to − 10 V, utilizing 14-bit DACs, achieving a precision of <85 mV after consideration of inaccuracies in the shift voltage and voltage drops across the PCBs [38] under all load conditions. Each channel can supply up to 20 mA. The bias currents are measured by 12-bit Analog-to-Digital Converters (ADCs) at an overall accuracy of <0.6%. Furthermore, the SBT can read eight Texas Instruments LMT01 temperature sensors. Two LMT01 are placed on the SBT board itself, while the remaining six can be connected externally, e.g., for MPPC temperature monitoring. We use an Atmel^TM^ ATMEGA328 micro-controller to manage ADCs, DACs, temperature sensor readout and to provide a serial interface to the FPGA on the associated SDIP. The SBT is powered from the bias power system, distributing the voltages via two SAFIR Bias Distribution (SBD) PCBs (see Figure 11a) to all SBTs.

We use a Keysight E3645A DC power supply for the shift-voltage and a Keysight E3646A dual Output power supply for the ±12 V. Both power supplies feature low ripple, as well as very good load and line regulation [39], required to achieve stable bias voltages for the MPPCs. The shift-voltage supply uses remote sensing and is connected by four 1.5 mm${}^{2}$, ∼12 m long wires. The ±12 V supply is connected by four wires using two for power and two for return.

#### 2.4.5. Power Supply, Conversion and Distribution

**Figure 12:**
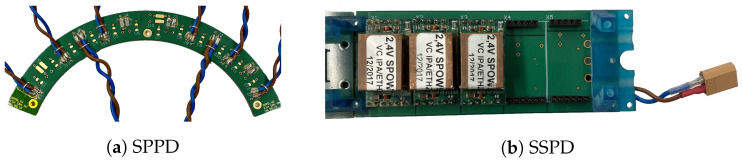
(**a**) The SPPD is responsible for distributing the main digital power. Two of these PCBs are part of SAFIR-I, each connecting the external power supply to 6 SSPD PCBs. (**b**) Each SSPD PCB distributes the digital power from the SPPD to the SDIP.

SAFIR-I requires five low voltages between 1.0 V and 3.3 V for its operation. The direct delivery of power at these low voltages from the power supply system to the PET insert located 10 m away is impractical because of the large wire cross section required in order to handle the resulting current of several hundred Amperes. We overcome this problem by using MRI-compatible DC-DC converter modules (SPOW) [19], developed in-house. Final conditioning of all low voltages is achieved by LDOs close to the consumers, powered from the output rails of the SPOWs. We feed a single 18 V line with ∼28.7 A to SAFIR-I and distribute it to all sectors using two SPPD PCBs (Figure 12a), which in turn connect to the 12 SSPDs (Figure 12b). A TDK Lambda Z20-40 EHFP (0–20 V/0–40 A, 800 W) power supply [40] is used as input and connected via a pair of ∼10 m long wires of 6 mm${}^{2}$ cross section, followed by a ∼ 1.3 m long section with 2.5 mm${}^{2}$ cross section. This cable reduction is necessary to meet the spatial constraints at the flange of SAFIR-I.

#### 2.4.6. Data Acquisition and Control

Communication with the insert, i.e., control and raw event data transfer, is achieved via 12 optical Ethernet links (1 Gbit·$s^{-1}$). Starting from the SFP modules on the SDIPs, six (double-) strands of optical fibers come together in one multimode breakout fiber cable (12 fibers), one per detector half. Two 10 m long multimode fiber cables run to the service room, where the same multimode breakout fiber cables connect them to three Intel X710DA4 network cards installed in the DAQ computer. All fiber cables are customized Fiberstore Optical Multimode 3 (OM3) Multimode Elite Trunk Cables with straight-through polarity.

The DAQ computer features a 16-core AMD Threadripper 2950x Central Processing Unit (CPU), 64 GB of Random Access Memory (RAM) and three Samsung EVO 970 2 TB Solid State Disks (SSDs) with a continuous write speed of 1500 MByte/s for data processing and short term data storage. Each SSD offers sufficient storage space for about 30 min of continuous data acquisition at 500 MBq (i.e., 90 min total). Beyond acquiring and storing event data arriving from SAFIR-I, the DAQ computer is also used to initialize, calibrate and configure the system, send and receive slow control data in order to control the SBT, and finally to control and monitor the power supplies, which are connected via Universal Serial Bus (USB) links.

Additional computing hardware for data analysis is connected using a 10 GBit/s link. Lastly, an auxiliary notebook is remotely connected to the DAQ computer, allowing the operator to control all hardware from within the MRI system control room. Both the DAQ computer and the operator’s notebook utilize a standard Linux distribution (Ubuntu 18.04).

### 2.5. DAQ Software

The DAQ software is comprised of several different modules, linked via Apache Thrift^TM^ [41]. One global settings file defines all detector parameters, thereby enabling the utilization of the same software for different setups, e.g., for testing.

The first module, called powerSupplyServer, handles the control and monitoring of all power supplies. After startup, the outputs are checked for short circuits and wrong polarity of the connections by applying 1 V and with a current limit of 70 mA in place. If the read back voltage is below 0.9 V, it is assumed that the reverse polarity protection diodes are limiting the output voltage. In this case, the software prevents that the power supply can be switched on at nominal power. Conversely, following a successful check the control is handed over to the user. As a safeguard against user error potentially causing damage to SAFIR-I, limit values for output voltage and current of all supplies are stored in the global settings file.

The second module, called EthernetServer, handles the reception and transmission of data to the scanner. During data acquisition, all incoming data is stored on one of the fast SSDs and command signals are sent to the hardware by means of this module. In order to distribute system calls and interrupt handling equally on the available CPU cores, the interrupt assignments are modified by the software and automatic interrupt balancing is disabled. The interrupt handler of each network interface is assigned to a different CPU core. Furthermore, the data processing threads, which handle the incoming data, are bound to the same cores that handle the interrupt routine. The incoming data is split into slow control data and measurement data and both are saved to different files for further analysis. Lastly, the EthernetServer continuously monitors the energy spectra and photopeak positions of selected channels (two per PETA6SE) to automatically adjust the bias voltage should a shift in the energy spectrum be detected.

The third module, called AsicControlServer, handles the configuration and calibration of the PETA6SE ASICs. The configuration parameters (741 values per PETA6SE) are stored in a single configuration file and usually remain unchanged between measurements. While most parameters are identical to all ASICs within a system, two parameters need to be individually calibrated for each PETA6SE channel to account for production tolerances; both calibration methods address physical properties in the components and therefore these steps only need to be performed after hardware components have been changed. The first parameter is the noise threshold of the discriminator in the front-end of the PETA6SE. To find the noise limit, the threshold value is systematically swept with deactivated bias voltage. Under these conditions the PETA6SE is triggered exclusively by electric noise; consequently, the noise threshold can be found by measuring the number of registered noise hits for different threshold values. During power up of the system, the timing threshold is set relative to the noise threshold (e.g., 60 mV above the noise value) to achieve a homogeneous detector response. The second parameter is related to all SiPMs, as well as all ASIC channels, having slightly different gains: The PETA6SE ASIC features the possibility to individually adjust the QDC gain of each input channel. Calibration of these gain values is performed through the automatic analysis of the recorded data from a reference source (${}^{22}$Na point source) positioned in the isocenter of the detector, for a constant bias voltage and different gain settings. The software selects specific gain values to receive matching energy spectra for all channels. Since the processing time of the ASIC correlates with the measured charge, this calibration ensures similar dead times in all channels.

The fourth module, called BiasControlServer, handles the communication with the SBT boards. During high-rate measurements, the power consumption of the SiPMs increases significantly (total bias current ≈0.8A), leading to a rise in the overall temperature and an increased break down voltage. In combination with ohmic losses in the cabling, this causes a change in the overvoltage of the SiPMs and ultimately a change of the SiPM gain. To counteract these effects, the bias voltage is adjusted automatically for each Detector Head Module (DHM). The data of 288 channels (two per ASIC) are constantly analysed by the EthernetServer, monitoring the photopeak position of the energy spectrum and comparing it with a reference value originally obtained during a low activity calibration measurement. The software then instructs the SBT to adjust the bias voltage so that the photopeak position is independent of the present activity or detector temperature. This allows us to use a count rate independent energy calibration during data processing.

Lastly, there are Graphical User Interfaces (GUIs) to allow for easy control of the entire system. They display the most important detector parameters and are used to interact with the hardware via the previously described software modules.

### 2.6. Data Calibration & Processing

The goal of data processing is to convert the measured raw data from the PETA6SE ASICs into list mode files for image reconstruction. During this process, different calibration data are applied and events are filtered by energy and timing windows.

#### 2.6.1. Calibration

The calibration data is generated in several steps. All calibration values are saved in a configuration file. They can be reused for subsequent measurements as long as the system has not been powered down, in which case timing related calibrations would need to be redone.

First, the QDC values *Q* are converted into deposited energy values by making use of the following formula:

(1) $$E=a_{i}\;\cdot \;{\left(Q-b_{i}\right)}^{c_{i}}.$$

The constants $a_{i}$, $b_{i}$ and $c_{i}$ are obtained individually for each channel *i* during the energy calibration. This has to be performed only once for a given hardware setup. It is based on three distinct points in the energy spectrum (0 keV, 340 keV (Compton edge) and 511 keV (photopeak)). The 0 keV point is obtained by deactivating the bias system and triggering the ASICs via test triggers. The other two points are obtained from a point source measurement. First we apply a Gaussian fit to the photopeak to get its position in the spectrum. Afterwards a combined fit of the photopeak and the Compton edge is used to obtain the last reference value.

Due to small manufacturing variations, the fine counter bin width is not constant for all 32 bins. Therefore, an individual time stamp for each bin is used, obtained from the fine counter calibration. For this calibration the distribution of fine counter bin values is observed after a point source measurement. The randomness of radioactive decay should result in an even distribution of fine counter values given equally wide bins, thus any deviation from such an even distribution indicates the true width of each bin. As these deviations are hardware dependent, the fine counter calibration values need to be acquired only once for a given setup.

Next, accurate time stamps must be obtained. The coarse counter is implemented in hardware through two linear-feedback-shift-registers. A lookup-table is accessed by software for the conversion of these counter values into sequential integer numbers. Combining the calibrated fine counter, the coarse counter and the epoch counter allows generating a 64 bit time stamp for each event. However, said time stamp still needs to be corrected for delays between the individual channels. These are caused by differences in the length of several signal lines, as well as inaccuracies in the sampling of the reset signal due to potential phase shifts between it and the PLL reference clock. This time delay calibration comprises two steps.

In the first step, the data are sorted for coincidences using a large coincidence timing window of 5 ns and the default energy window (391–601 keV). Prior to calibration, the channels are grouped by the associated TDC in the ASICs. For each TDC pair i,j, the mean value of the timing distribution $m_{ij}$, the standard deviation $s_{ij}$, and the number of coincidences $n_{ij}$ are measured. Then, we minimize the following equation:

(2) $$\sum_{ij}\frac{{\left(m_{ij}-d_{i}+d_{j}\right)}^{2}}{s_{ij}^{2}\;n_{ij}}$$

with the delay $d_{x}$ corresponding to the TDC *x*.

In the second step, the data set is analyzed again, with the TDC delays already applied and a reduced coincidence window of 1.5 ns. The same calibration routine is used but this time for the individual channels instead of channel groups.

The last calibration to be performed compensates for the time walk effect, which is modeled by:

(3) $$\Delta t=\lambda {\left(E-511\;\mathrm{keV}\right)}^{2}.$$

The constant λ is obtained by analyzing the data set with a large energy window (211–661 keV) and a 1.5 ns coincidence timing window, while applying all calibration data. The coincidence events are filtered further by requiring that one of the hits is within 461–561 keV. We analyze the time difference between the two hits in every coincidence event for different energies and obtain the calibration constant λ by fitting to the distribution.

#### 2.6.2. Data Processing

Following the application of the above-described calibrations to the raw data, the calibrated events comprise hits with a time stamp in pico seconds, an energy in keV, as well as a ring number and crystal number encoding the exact location of detection in SAFIR-I. A first energy window is applied (usually 391–601 keV, or 100–610 keV if inter-crystal scatter recovery is enabled) and the hits are sorted in time. Afterwards, we use a single window coincidence sorter (typically with a 500 ps CTW) to find the coincidence events in this data stream.

At this stage, an optional inter-crystal scatter recovery [27] can be performed. If enabled, hits within a certain radius are considered as scattered interactions and the software combines these hits to one event, by adding their energies together and using the energy weighted average of their time stamps. At the end of this process, the recovered hits still have to pass through our standard energy window (usually 391–601 keV).

Next, coincidences with more than two hits are removed and a cut on the minimum tangential angle between the hits is applied. The latter effectively limits the FOV to a cylinder with a diameter of ∼85 mm. The remaining coincidences are saved in a list mode file for image reconstruction.

### 2.7. Image Reconstruction

The list mode files containing processed coincidences are reconstructed using a custom version of the Software for Tomographic Image Reconstruction (STIR) library [42]. In contrast to the standard library, the exact geometry of the SAFIR-I PET system is used, instead of a conventional cylindrical approximation. The improvements are described in detail by Khateri et al. [43].

An absolute quantification of voxel values can be achieved through the reconstruction of PET data collected with a cylindrical calibration phantom, in the approximate size of the object to be studied (e.g., a rat) and at a known, homogeneous decay rate concentration. For best accuracy, random, attenuation, scatter and normalization corrections should be included in the reconstructed image, as described by Khateri et al. [32]. A calibration factor for the quantification can then be found by comparison of the average voxel value in the reconstructed image (in arbitrary units) with the expected decay rate in the voxel volume (in MBq·${\mathrm{cc}}^{-1}$).

### 2.8. Measurements

Several measurements have been made with SAFIR-I to gauge its MRI compatibility, performance and aptitude for preclinical research under real conditions.

#### 2.8.1. Interference of SAFIR-I with the MRI System

The compatibility of the SAFIR insert with the Bruker BioSpec 70/30 USR MRI system was assessed using a dedicated cylindrical water phantom (Bruker BioSpin MRI PHAN 1H IM RAT HEAD, model no. 1P T10475, Bruker Corporation, Billerica, MA, USA), a suitable, tunable and matchable transmit/receive coil (Bruker BioSpin RF RES 300 1H 075/040 QSN TR, model no. 1P T13161V3, Bruker Corporation, Billerica, MA, USA) and the associated interference measurement procedure. The procedure combined an adjustment of the static magnetic field, active shimming of the phantom region, Bruker’s Echo-Planar-Imaging (EPI) based quality assurance routine *EPI QA1* and finally a fully automated determination of the SNR. The SNR measurement was repeated five times, allowing for the estimation of mean value and standard deviation.

Following the described procedure, five measurements were made in immediate succession:

- Baseline: the MRI system alone, without SAFIR inserted.
- Unpowered: with SAFIR inserted but not powered at all.
- Powered: with SAFIR inserted and fully powered, bias voltages off.
- Readout-ready: with SAFIR inserted and fully powered, bias voltages on.
- Readout: with SAFIR inserted and fully powered, while actively acquiring and transmitting event data.

For ‘Readout’, data were acquired from a ${}^{22}$Na point source (400 kBq); it was placed at the tip of the Bruker phantom.

Furthermore, possible effects on the MRI system during a high decay rate imaging were studied. To that end, two additional studies were conducted:

- Baseline: SAFIR (fully powered) inside the static magnetic field of the MRI system.
- High-rate test: SAFIR actively acquiring and transmitting event data from a high-activity source.

For the high-rate test, a small glass vial containing ∼1.3 mL of an ${}^{18}$F in water solution (decay rate range over the measurement duration 325.7–351.3 MBq) was used; it was again placed at the tip of the Bruker phantom.

#### 2.8.2. Interference of the MRI System with SAFIR-I

In a second step, possible interference on SAFIR-I caused by the MRI system was assessed. To that end, a ${}^{22}$Na point source (365 kBq) was imaged under varying conditions and the CRT and energy resolution were recorded in each case. Additionally, a count value was obtained for each measurement, by normalizing the recorded number of coincidences with the expected number of positron emissions from the source and comparing that value to the baseline count value (defined as 1). To get a baseline, the source was placed in SAFIR-I’s isocenter, together with a water-filled 15 mL Falcon tube acting as phantom for the MRI system. The same transmit/receive coil as before (see Section 2.8.1) was used, surrounding the water phantom and point source. The point source was then imaged for 100 s inside the MRI system’s static magnetic field $B_{0}$. The imaging process was subsequently repeated with one of the following four MRI sequences running simultaneously: T1-FLASH, T2-TurboRARE, EPI-LR (axial, left to right), EPI-HF (coronal, rostral to caudal).

Separately, the potential effects on high-rate measurements were analyzed. For this test, a 50 mL Falcon tube, filled with a solution of ${}^{18}$F in water (decay rate range over the experiment duration 491.7 MBq–521.1 MBq) was used. Data was again acquired with SAFIR-I placed in the MRI system’s static magnetic field, and while running each of the four described MRI sequences. Accounting for the different sequence durations, in an attempt to maximize the number of measurements at the highest activities, the execution order for the high decay rate experiment was: EPI-LR, EPI-HF, T1-FLASH, T2-TurboRARE, static $B_{0}$. The acquisition time was 10 s for each measurement.

#### 2.8.3. Coincidence Energy Resolution, Coincidence Resolving Time and Estimated Peak Sensitivity

By means of a point source (${}^{22}$Na, 362 kBq) measurement, coincidence energy resolution, CRT and coincidence detection efficiency, i.e., the relative amount of detected coincidences compared to the expected number of positron emissions, were obtained at low decay rate. The source was positioned in the isocenter of SAFIR-I and imaged for 60 s. To gauge the scanner’s performance at high decay rates, a ${}^{18}$F (in water) line source of 75 mm length and 4.5 mm diameter was imaged for 10 s at 551.6 MBq, again determining the CRT and energy resolution. The line source was aligned with the central axis of SAFIR-I and centered with respect to the axial FOV. The scanner was freshly calibrated before taking either of these measurements. For the analysis of both data sets, an energy window of 391–601 keV was used, and the Coincidence Timing Window (CTW) was set to 500 ps. No data corrections (e.g., random or scatter corrections) were applied.

The energy resolution was then found as the FWHM of the photopeak in the coincidence energy spectrum, determined by a Gaussian fit on the peak between 480–580 keV. No rationale exists, a priori, to predict the exact width of the coincidence time spectrum peak. Hence we used a double fit method, employing a first fit to determine the approximate peak position and standard deviation σ, before performing a second, more exact fit between −2σ and +2σ, which in turn produced the CRT as its FWHM value. Lastly, the coincidence detection efficiency observed with the point source data set could be used to estimate SAFIR-I’s peak sensitivity value. A comparable evaluation at high decay rate was not possible, due to the geometry of the line source spanning the entire FOV and even extending beyond it.

#### 2.8.4. Truly Simultaneous PET/MR Imaging of a Rat Brain In Vivo

The brain of a Sprague Dawley rat was imaged in vivo with SAFIR-I. We imaged in two steps, using ${}^{18}$F-Fluorodeoxyglucose (FDG). First, 29.5 MBq were injected into the femoral vein of the animal and data were acquired continuously for 45 min. Then, the decay rate was increased for a second injection, bringing the total injected activity to 314.6 MBq, and the brain was imaged continuously for another 45 min. The PET image acquisition was started immediately after injection. An 84 s gap separated the two imaging blocks.

As part of the reconstruction, the rat imaging blocks were cut into time frames of desired duration. This was done twice: First, we cut both the low and high decay rate data set according to a scheme exemplary for planned preclinical studies with SAFIR-I (10 × 30 s, 5 × 60 s, 5 × 120 s, 5 × 300 s). Second, we extracted a 5 s frame from the high decay rate data set to get an indication on image quality during planned high-speed acquisitions. All frames were then reconstructed without any data corrections using STIR, utilizing the iterative Ordered Subset Expectation Maximization (OSEM) algorithm with 10 subsets & 30 iterations, the SAFIR-I geometry and inter-iteration Gaussian filtering (FWHM 1.1 mm × 1.1 mm × 2.2 mm). Based on features visible in the reconstructed images, in particular the cerebral cortex, an estimate on the achieved spatial resolution was made.

Together with the PET imaging, we also recorded several MR images, in a truly simultaneous PET/MRI acquisition.

## 3. Results

In the following, we summarize the results from the measurements described above.

### 3.1. Interference of SAFIR-I with the MRI System

The results in terms of mean SNR achieved in each study are summarized in Table 1 and Table 2 below. All recorded SNR values are well above the minimum value of 2000 m$m^{-3}$ guaranteed by the manufacturer for the MRI system used. The observed maximal deviations from the baseline are 3.6% and −2.44%, for the low and high decay rate, respectively.

**Figure 13:**
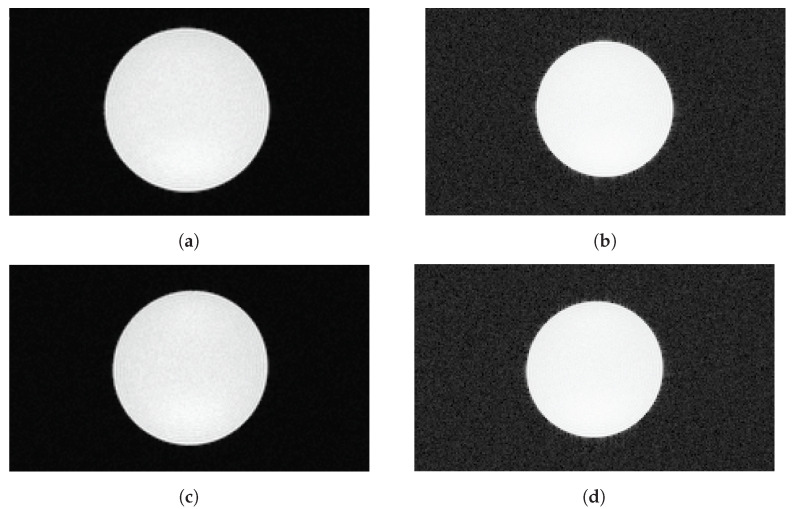
MR images of a cylindrical water phantom to determine SNR (transverse view). Images taken in parallel to data recording with the SAFIR insert inside the MRI machine during readout at low decay rate (top row) and during the high-rate test (bottom row). (**a**) Standard image, readout, (**b**) Modified greyscale version, readout, (**c**) Standard image, high-rate test, (**d**) Modified grayscale version, high-rate test.

No unexpected artifacts could be seen in any of the images. Example SNR images are shown in Figure 13a–d. The cylindrical cross section of the water phantom is clearly visible with sharply defined edges. Modified grayscale versions of the standard images show there are no artifacts hidden in the background either.

### 3.2. Interference of the MRI System with SAFIR-I

The effects of running different MRI sequences on key SAFIR-I performance parameters in simultaneous PET data acquisition, from the point source at low and Falcon tube at high decay rate, are summarized in Table 3 and Table 4, respectively.

### 3.3. Coincidence Energy Resolution, Coincidence Resolving Time and Peak Sensitivity

The results for the point source (362 kBq) and line source (551.6 MBq) measurements are summarized in Table 5.

Based on the coincidence detection efficiency in the low decay rate measurement, the estimate of the peak sensitivity value is (1.649±0.003)%.

### 3.4. Truly Simultaneous PET/MR Imaging of a Rat Brain In Vivo

**Figure 14:**
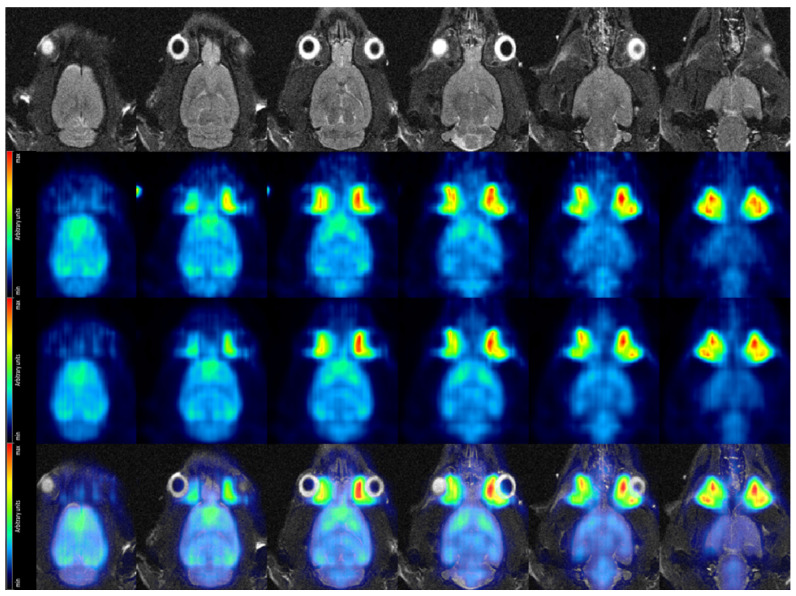
Coronal view of the rat brain in vivo. (**Top row**): MR images acquired with a T2-TurboRARE sequence. (**Second row**): PET data acquired at low dose. Average over frames 22–25, corresponding to 25–45 min after tracer injection. (**Third row**): PET data acquired at high dose. Average over frames 22–25, corresponding to 25–45 min after tracer injection. (**Bottom row**) Fused MRI and high dose PET data.

Figure 14 shows a selection of coronal view cuts of the rat brain for both high and low decay rate, the MR slices recorded with a T2-TurboRARE sequence serving as anatomical reference and finally the fused PET/MRI images (for the high decay rate measurement). Visualization of PET data and the fusion of PET and MR images were achieved using the software PMOD [44]. Particularly in the PET images acquired at high decay rate, the horseshoe-shaped cerebral cortex, which typically presents a thickness of 1.5–2.5 mm in rats [45], is clearly distinguishable.

**Figure 15:**
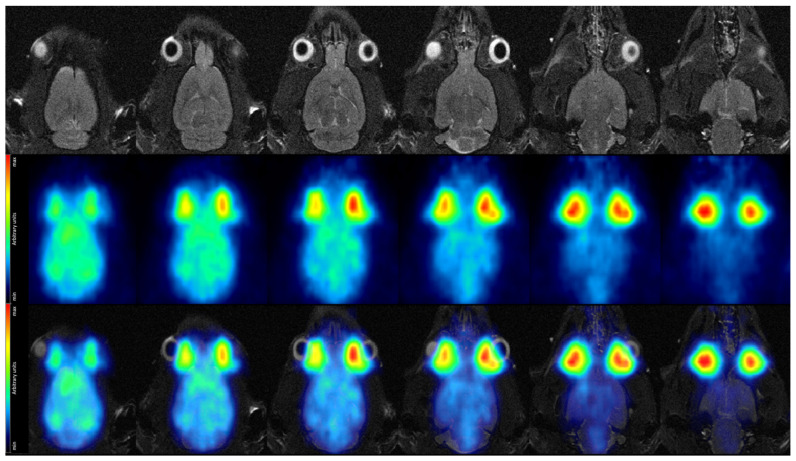
Coronal view of the rat brain in vivo. (**Top row**): MR images acquired with a T2-TurboRARE sequence. (**Middle row**): PET data acquired at high decay rate in a single 5 s time frame, recorded 44 min after tracer injection. (**Bottom row**): Fused MRI and high-rate PET data.

Similarly, we depict the PET image obtained from the 5 s time frame at high decay rate in Figure 15, again showing the T2-TurboRARE MR image and the fusion between the two in addition.

## 4. Discussion

In case of an incompatibility of SAFIR-I with the MRI system, the MR images would show obvious artifacts, indicative of induced eddy currents. Additionally, a severe degradation of the latter system’s SNR compared to the baseline measurements would be expected [46,47]. When looking at the effects of operating our SAFIR-I PET insert simultaneously with the MRI system, only few-percent deviations in the latter’s SNR values compared to baseline measurements could be observed in all studies. In the low decay rate tests, the small apparent increase of 3–4% in test conditions compared to the baseline measurement was likely caused by a minute positioning variation of the transmit/receive coil inside the system, at the time of installation of SAFIR-I inside the bore. In the case of the second measurement, carried out at high decay rate, small coil positioning variations and a slight increase in noise caused by SAFIR-I are equally possible sources of the ∼2.5% decrease in SNR. Nevertheless, the overall minuscule fluctuations in SNR of the MRI system, together with the absence of visible artifacts in the SNR images, indicate that the operation of SAFIR-I, even at high decay rates, does not hinder simultaneous MR image acquisition.

The other way around, when the focus was set on the performance of the PET insert while running different MR sequences, mostly values were observed to be, either fully compatible with the respective baseline, or fluctuated from the baseline values by mere per mil. The latter can be explained by random errors between the different measurements. The only exception from this rule are the results obtained for energy resolution at high decay rates (fluctuations ≲ 2 ). The energy resolution is susceptible to degradation if an increased number of random coincidences, e.g., between pairs of similarly attenuated photons, occur. The random rate in turn is proportional to the decay rate. The observed fluctuations in energy resolution can thus be attributed to the changing contribution from randoms in the recorded data over the range of decay rates at which the different measurements were made. In fact, the improvements in measured energy resolution follow exactly the reduction in decay rate, i.e., the order in which the single experiments were carried out.

The contribution from randoms also explains why the energy resolution, already for the baseline value, is worse at high compared to low decay rate. In the case of the CRT, the larger diameter of the active region in the phantom compared to the point source enhances the divergence between the values reported at different rates. With all observations justifiable, it can be concluded that SAFIR-I is immune to possible adverse affects of the MRI system. Hence, compatibility between the two devices is ensured in both ways.

Looking at the performance parameter measurements, we find that SAFIR-I offers adequate energy resolution and sensitivity. With respect to CRT, we achieved our ambitious target value of ∼300 ps at 500 MBq. Moreover, the values in Table 5 demonstrate that SAFIR-I is capable of outperforming this value by ∼30% towards lower decay rates. This striking result may enable us in the future to operate our detector also at lower decay rates than originally foreseen, using TOF-PET, i.e., the associated SNR gain factor [7,48], to compensate the signal reduction resultant of identically short time frames at decreased decay rate. Accurate estimates on the final performance at different decay rates will however only be possible, after we integrate all previously mentioned data corrections in the reconstruction of SAFIR-I data.

Beyond the absolute magnitude of the reported performance parameters (Section 3.3), it can be observed that CRT and energy resolution are slightly better compared to the results reported in Section 3.2, with the exception of the high decay rate value for the energy resolution. This is partly due to the deliberate care which has been put into the calibration of SAFIR-I for these performance measurements, whereas for the interference measurements only relative changes in detector performance were of interest. The values for CRT at high decay rate additionally profit from a smaller source diameter of the line source compared to the Falcon tube. The energy resolution at high decay rate, decreased by ∼1.5–2% compared to the values reported in Section 3.2, can be explained by the higher decay rate at which this performance measurement was taken (551.6 MBq vs. ≤ 521.1 MBq).

Summarizing the in vivo rat brain study, an improvement on image quality from the low to the high dose acquisition (second and third row of Figure 14) is apparent. The clearly discernible cerebral cortex demonstrates that we have achieved our targeted spatial resolution of ∼2 mm. Once we will have implemented data corrections to the reconstruction of SAFIR-I data and obtained a measurement with an appropriate calibration phantom, we will be able to quantify the image voxels and show Time-Activity Curves (TACs) for the rat’s cerebral cortex at both low and high decay rates.

The image reconstructed from a single 5 s time frame out of the high-rate imaging block (Figure 15) gives a first indication on image quality at high acquisition speed, while finer details are partially obscured, more prominent structures are still discernible. The cortex, although less clearly delineated, is still recognizable in most slices, particularly with the MR image at hand providing anatomical reference. However, SAFIR-I’s true high-speed acquisition capability will become apparent only when calculating TACs, where it could enable us to show the rising edge of a TAC with unprecedented temporal resolution.

Regarding the MR images depicted in Figure 14 and Figure 15, it’s worth mentioning that the image quality was not optimal; this was not caused by SAFIR-I but by a small (∼1–2 cm), inadvertent axial offset between the rat’s head and the center of the MRI system in this measurement.

In summary, these results demonstrate that SAFIR-I can be used as a tool for fast kinetic preclinical studies, as intended. Proof of SAFIR-I’s indicated quantitative abilities necessitates measurements with calibration phantoms of known volume and decay rate, in addition to requiring an adaptation of the current reconstruction procedure for SAFIR-I data, to include all data corrections previously implemented for the prototype system.

## 5. Conclusions

We successfully tested the SAFIR-I system and could show that it fulfills the design criteria set for the SAFIR project. In particular, our PET insert is MR-compatible and delivers the required energy, timing and spatial resolution. Furthermore, we could demonstrate that dynamic preclinical studies, relying on truly simultaneous acquisition of PET/MRI, are possible with SAFIR-I.

In the months to come, SAFIR-I will be fully characterized according to the NEMA Standards Publication NU 4-2008 [28] standard. In the course of the characterization, random, attenuation, scatter and normalization corrections will be included in the SAFIR-I data reconstruction. Additionally, we will acquire the necessary data from cylindrical phantoms of standardized size to determine activity-dependent decay rate concentration calibration factors, enabling fully quantitative PET imaging in rats and mice. Furthermore, we intend to successfully perform a first ${}^{15}$O-tracer based study.

Simultaneously, the SAFIR collaboration will complete the construction of the so-called full detector, Small Animal Fast Insert for MRI detector II (SAFIR-II), featuring comparable architecture but 145 mm axial coverage, thereby offering full-body small animal imaging capabilities. Furthermore, the increased axial coverage will augment the sensitivity. We expect a system sensitivity averaged over the entire FOV of 4% using inter-crystal scatter recovery.

These performance gains will eventually allow even shorter time frames of the order of ∼1 s, thereby increasing the image acquisition speed.

## Figures and Tables

**Table 1:** SNR values observed in baseline and interference measurements (low decay rate).

Condition	SNR [ m$m^{-3}$]	Deviation from Baseline [%]
Baseline	2532 ± 6	–
Unpowered	2622 ± 8	+3.6 ± 0.4
Powered	2607 ± 9	+3.0 ± 0.4
Readout-ready	2614 ± 14	+3.2 ± 0.6
Readout	2623 ± 12	+3.6 ± 0.5

**Table 2:** SNR values observed in baseline and interference measurement (high decay rate).

Condition	SNR [ m$m^{-3}$]	Deviation from Baseline [%]
Baseline	2502 ± 4	–
High-rate test	2441 ± 7	−2.44 ± 0.32

**Table 3:** CRT, energy resolution and count value (observed coincidences normalized by expected counts, relative to baseline) in low decay rate (365 kBq) PET data acquisition during simultaneous MRI operation.

Condition	CRT	Energy Resolution	Count Value
Static B${}_{0}$ (baseline)	(216.8±0.3) p s	(12.67±0.02)%	1
T1-FLASH	(216.7±0.3) p s	(12.65±0.02)%	0.994±0.002
T2-TurboRARE	(216.9±0.3) p s	(12.66±0.02)%	0.998±0.002
EPI-LR	(216.6±0.3) p s	(12.64±0.02)%	0.994±0.002
EPI-HF	(216.8±0.3) p s	(12.61±0.02)%	0.998±0.002

**Table 4:** CRT, energy resolution and count value (observed coincidences normalized by expected counts, relative to baseline) in high decay rate (range 491.7–521.1 MBq) PET data acquisition during simultaneous MRI operation.

Condition	CRT	Energy Resolution	Count Value
Static B${}_{0}$ (baseline)	(382.9±0.2) p s	(18.819±0.009)%	1
T1-FLASH	(385.2±0.2) p s	(19.00±0.01)%	0.9970±0.0003
T2-TurboRARE	(384.0±0.2) p s	(18.97±0.02)%	0.9998±0.0003
EPI-LR	(385.5±0.2) p s	(19.21±0.01)%	0.9998±0.0003
EPI-HF	(385.2±0.2) p s	(19.13±0.01)%	0.9996±0.0003

**Table 5:** CRT and energy resolution at low and high decay rate with the point and line source, respectively.

Parameter	Low Decay Rate Value	High Decay Rate Value
CRT	(209.0±0.3) p s	(326.89±0.12) p s
Energy resolution	(12.41±0.02)%	(20.630±0.011)%

## Data Availability

Data generated in the SAFIR project is part of the general project results. As such, all data are legally owned by the respective employers of the researchers involved, ETH Zurich and the University of Zurich, and will not be shared.

## Abbreviations

The following abbreviations are used in this manuscript:

ADC	Analog-to-Digital Converter
APD	Avalanche Photo Diode
ASIC	Application Specific Integrated Circuit
CBF	Cerebral Blood Flow
CNC	Computerized Numerical Control
CPU	Central Processing Unit
CRT	Coincidence Resolving Time
CT	Computed Tomography
CTW	Coincidence Timing Window
DAC	Digital-to-Analog Converter
DAQ	Data Acquisition
DHM	Detector Head Module
DOI	Depth Of Interaction
dSiPM	digital SiPM
EPI	Echo-Planar-Imaging
FDG	^18^F-Fluorodeoxyglucose
FOV	Field Of View
FWHM	Full Width at Half Maximum
FPGA	Field-Programmable Gate Array
GATE	GEANT4 Application for Emission Tomography
G-APD	Geiger-mode APD
GUI	Graphical User Interface
IC	Integrated Circuit
ICSR	Inter-Crystal Scatter Recovery
LC	Lucent Connector
LDO	Low Dropout Regulator
LGSO	Lutetium Gadolinium Oxyortho Silicate
LSZH	Low Smoke Zero Halogen
LVDS	Low Voltage Differential Signaling
LYSO	Lutetium Yttrium Oxyortho Silicate
MLEM	Maximum-Likelihood Expectation-Maximization
MPPC	Multi Pixel Photon Counter
MR	Magnetic Resonance
MRI	Magnetic Resonance Imaging
MTP	Multi-fiber Termination Push-on/Pull-off
NECR	Noise-Equivalent Count Rate
NEMA	National Electrical Manufacturers Association
NEMA-NU4	NEMA Standards Publication NU 4-2008
OSEM	Ordered Subset Expectation Maximization
OM3	Optical Multimode 3
PCB	Printed Circuit Board
PET	Positron Emission Tomography
PETA6SE	Position-Energy-Timing Application Specific Integrated Circuit, version 6, Single Ended
PLL	Phase-Locked-Loop
QDC	Charge-to-Digital Converter
RAM	Random Access Memory
RF	Radio Frequency
SAFIR	Small Animal Fast Insert for MRI
SAFIR-I	Small Animal Fast Insert for MRI detector I
SAFIR-II	Small Animal Fast Insert for MRI detector II
SBD	SAFIR Bias Distribution
SBT	SAFIR Bias and Temperature
SDIP	SAFIR Digital Interface Board PETA
SFCD	SAFIR Fast Control Distribution
SFCM	SAFIR Fast Control Master
SFP	Small Form-factor Pluggable
SiPM	Silicon Photomultiplier
SNR	Signal-to-Noise Ratio
SPOW	SAFIR Power Converter
SPPD	SAFIR Primary Power Distribution
SP6	SAFIR PETA6 Board
SSD	Solid State Disk
SSPD	SAFIR Secondary Power Distribution
STIR	Software for Tomographic Image Reconstruction
TAC	Time-Activity Curve
TDC	Time-to-Digital Converter
TOF	Time-of-Flight
UDP	User Datagram-Protocol
UPC	Ultra Physical Contact
USB	Universal Serial Bus
USR	Ultra Shield Refrigerated
